# Address of Wm. O. Kulp, D.D.S

**Published:** 1867-08

**Authors:** Wm. O. Kulp


### ADDRESS.

BY WM. O. KULP, D. D. S.

Read before the Iowa State Dental Society.

*Gentlemen of the Iowa State Dental Society:*

I have thought it would not be amiss at this time to recall some of the workings of our Society as well as its history. It may do us good, in that it will aid us to see that we have REALLY made advancement in our standing as a Profession, as well as in personal abilities; and thus lead us to go on and upward the Hill of Science, until we shall be masters of our position. May it be so *is* my prayer!

A few years ago a few members of our Society, conceived the idea of calling a State Dental convention, to consider Dental matters; also to organize a State Society, if possible. After writing many letters to different members of our profession throughout the State, a call for a convention was issued to assemble at Muscatine, July 14th, 1863, to which six members of the profession answered; after considerable discussion and "cross-firing," a resolution was passed, declaring that we would organize a State Dental Society. A committee on permanent organization was appointed, whose report was adopted, and our worthy Bro., Dr. Chase, then of Independence, was elected first President, and Dr. McCarvey from Dewitt, Recording Secretary.

Still at this fair start, the opposition to its future growth by those who ought to have been its friends, was not without great effort, as letters written (which have since come to light) would show; but another call was issued for a meeting to take place at Iowa City, January, 1864, which was responded to more liberally, as we had at that meeting eleven members present. Here a constitution and order of exercises were adopted. Dr. Tulloss elected presiding officer, our Recording Secretary was re-elected, and your humble servant was elected Corresponding Secretary. The meeting was so full of the good spirit, and all profited so much, that we felt that the next annual meeting must be a perfect success; and so it was, as the meeting at Davenport, in July following showed, where there were present twenty members of the profession. This meeting will ever be a bright spot in the history of our society; those of us who were present, all feel that we grew much in professional stature those few days. The start we got then we have shown in our daily practice, was one in the right direction; how many remember that the use of the mallet and wedge were among the good things served up at that feast, as well as that heroic treatment of abscess, which has so characterized our success in that direction ever since; and many more things of no lesser good to us and our suffering patients. Compare days prior to this meeting, with our standing now; and can any one say our Society has done us no good. At this meeting the old officers were re-elected, and another semi-annual meeting was ordered to come off at Des Moines in January, 1865, at which still more names were added to our membership; much was done at this meeting to bring our State organization to a level, if not above any similar one. On account of the difficulty of reaching Des Moines during the winter, there were many of the old members absent, and we decided to discontinue our winter meeting for a time at least.

Dr. Ingersoll was elected President; Dr. Chase, Recording Secretary; Corresponding Secretary was re-elected. Our next meeting was held in Dubuque, in July following, and we had one of our largest gatherings; many new members were added, much good was done to the profession in Iowa, and the bonds of Society were very much strengthened. It was a great benefactor to the deciduous teeth, as that subject was ably discussed, and confessions were made and amends promised, that if adhered to, much has been accomplished in the right direction.

The old officers were re-elected. The next annual meeting was held at Burlington, July, 1866, at which was one of our best meetings; and many new names were added to our membership; and some of the most important as well as scientific subjects were very ably written upon and discussed. It is quite interesting to compare the nature of our discussions here with those of our first meetings.

Instead of discussing fees and rubber, and imagining what was the best way to prevent saliva or mucus from running down the necks of teeth, and thus interfering with filling cavities in superior incisors, as we did at Iowa City; we were enabled to stand alone as it were, and look into the mysteries of Dental Physiology, Pathology, Hygiene, and Chemistry: and this year we are to go still further in our discussions, judging from the bill of fare served by our noble Corresponding Secretary. Looking over the past, and to the present, can we say that the Iowa State Dental Society was formed in vain? Has it done no good? Yea, verily, and may it still go on until the time is that he who does not attend its meetings and profit thereby, "Shall be despised as one neglecting his duty, to his patients and his profession, and both unite to sting him out of it without mercy;" and judging from the past, let us take new courage and strength, and go on doing our whole duty, calling on science to aid us in our investigations after truth, until we have unfolded all her mysteries, and we are truly masters of our chosen profession; and let us encourage Dental education in every way, let us not take any one into our profession who is not willing to fully conform with the precedence set forth by the committee appointed by the American Dental Association at its last meeting on this subject—two years of Office tuition and two full courses of lectures at some regular Dental College—and let us as preceptors see to it, that our students are FITTED for College. The country is overrun with Dentists, so-called, who are degrading our profession and are fast bringing it down to a trade level; and the fact is, Dentists are to blame for it, for how many of us have turned out six months' students on to the public; (I am thankful to say, I am not guilty of this error, if I am ever so faulty in other things); all ought to have more love for our institutions of Dental education; many who are still without their honors who are worthy, who ought to enjoy them; such ought to show themselves at such places, and make known their wants, and submit to an examination, and receive the honor which shall show him to be a regular practitioner of Dentistry; and by example, tell those coming after him, that he honors these blessed influences coming from these institutions, and he can then CONSISTENTLY urge our students to go through a regular course of study. Gentlemen! these institutions are monuments in our profession, and they need our unqualified support as well as we need their benign shadows to fall around us; and let me again urge you to take no one into our profession, who will not go through an entire course of study in one of these, and graduate. Then we shall continue to advance our noble profession, even after we are gone to our long home. In conclusion, let me urge all who are here present to-day; be not wanting in zeal and earnestness in our search after truth; let us get and give the best there is, and not criticise words during our discussions; but freely do so with error, and let us not lose sight of the fact, that as our Society stands, so does the reputation of the profession in our great State; let us be dignified, earnest, honest and true, then all will be well. I thank you, gentlemen, for your assistance and courtesies to me during my reign as your president, and let us not forget to be thankful to Him who holds us all in the hollow of his hand, for his many blessings to us individually, since our last meeting, and for this propitious gathering to-day. May He still continue His favors to us all.

